# Supplementary material for: MiR-218-5p/EGFR Signaling in Arsenic-Induced Carcinogenesis
Source: Cancers (Basel). 2023 Feb 14;15(4):1204. doi: 10.3390/cancers15041204 (PMC9954652; doi:10.3390/cancers15041204)
Supplement: Supplementary file 1 [file cancers-15-01204-s001.zip › cancers-2219516-supplementary.pdf]

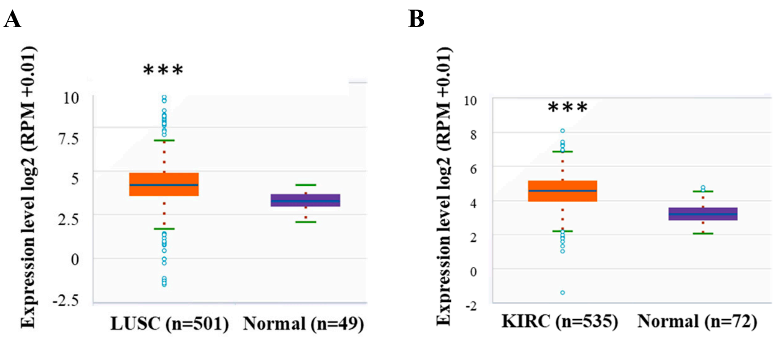

Figure S1. EGFR is highly expressed in (A) lung squamous cell carcinoma (LUSC) and (B) Kidney Renal Clear Cell Carcinoma (KIRC) on publicly available datasets from TCGA (cancer tissues vs. normal) and following the relevant algorithms (https://starbase.sysu.edu.cn/, accessed on 12 October 2022). *** $p < 0.001$ compared to normal tissues.

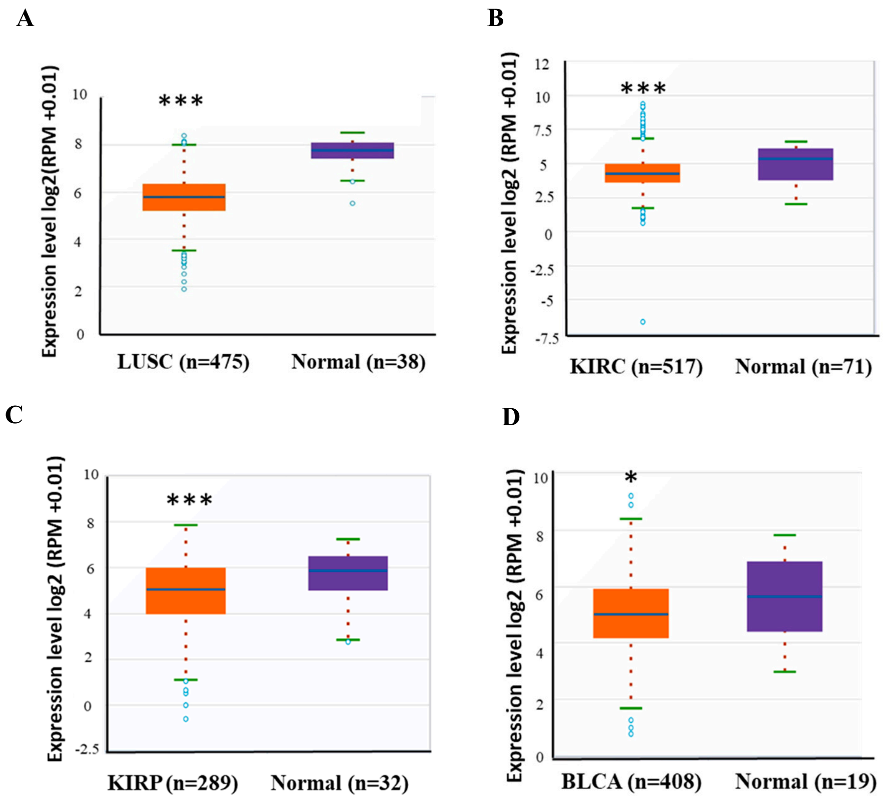

Figure S2. The expression levels of miR-218-5p in (A) lung squamous cell carcinoma (LUSC) and (B) Kidney Renal Clear Cell Carcinoma (KIRC) (c) Kidney Renal Papillary Cell Carcinoma (KIRP) and (D) Bladder Urothelial Carcinoma (BLCA) on publicly available datasets from TCGA (cancer tissues vs. normal) and following the relevant algorithms (https://starbase.sysu.edu.cn/, accessed on 12 October 2022). * $p < 0.05$ and *** $p < 0.001$ compared to normal tissues.

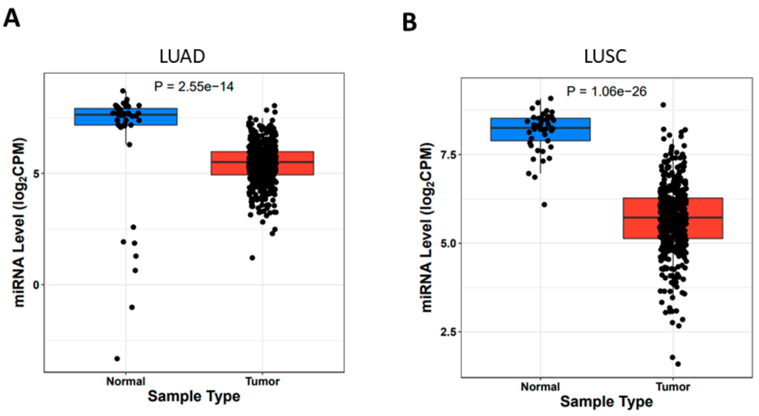

Figure S3. The expression levels of miR-218-5p in (A) lung adenocarcinoma (LUAD) and (B) lung squamous cell carcinoma (LUSC) on publicly available datasets from TCGA (normal Vs. tumor) and following the relevant algorithms (http://bioinfo.jialab-ucr.org/CancerMIRNome/, accessed on 29 January 2023).

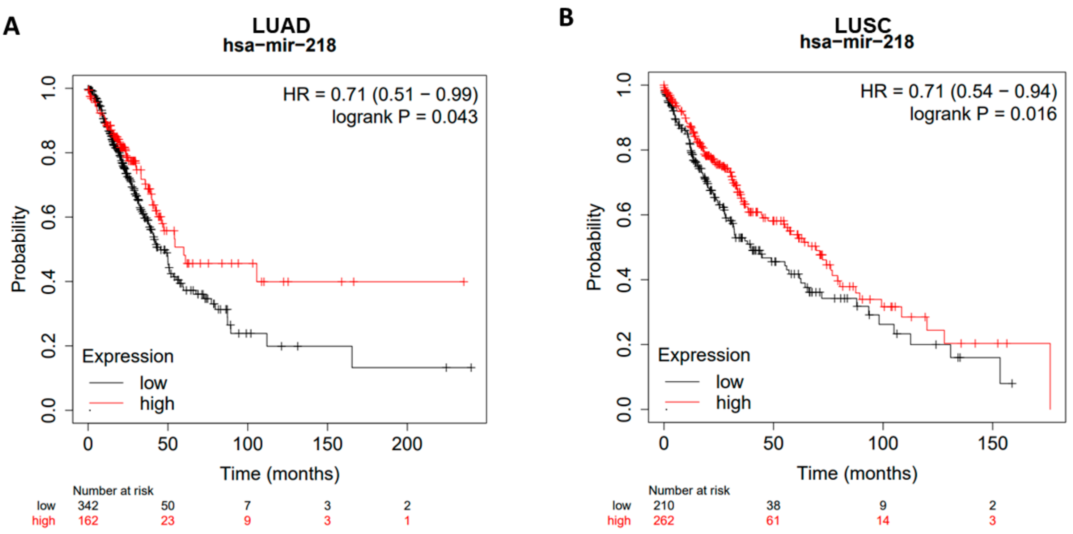

Figure S4. Kaplan–Meier plots for progression-free survival probability with lung cancer patients classified according to low and high miR-218-5p in (A) Lung Adenocarcinoma (LUAD) (A) and lung squamous cell carcinoma (B) (LUSC) on publicly available datasets from TCGA (low vs. high) and following the relevant algorithms (https://kmplot.com/analysis/index.php?p=service, accessed on 29 January 2023).
